# The orbitofrontal cortex modulates parenting stress in the maternal brain

**DOI:** 10.1038/s41598-018-38402-9

**Published:** 2019-02-07

**Authors:** Madoka Noriuchi, Yoshiaki Kikuchi, Kumiko Mori, Yoko Kamio

**Affiliations:** 1 Department of Frontier Health Science, Division of Human Health Science, Graduate School of Tokyo Metropolitan University, 7-2-10 Higashi-ogu, Arakawa, Tokyo 116–8551 Japan; 2 National Institute of Mental Health, National Center of Neurology and Psychiatry, 4-1-1 Ogawahigashi, Kodaira, Tokyo 187–8553 Japan; 3 Institute of Education and Human Development, Ochanomizu Univesrsity, 2-1-1 Otsuka, Bunkyo, Tokyo 112–8610 Japan

## Abstract

Many mothers are adaptive, deploying successful coping strategies that mitigate the deleterious effects of parenting stress on caregiving, nevertheless, the neural mechanisms underlying these adaptive responses remain unclear. We utilized functional magnetic resonance imaging to investigate brain activity in 28 healthy mothers of typically developing, 2-to-3-year-old children in response to the feeding behavior of their own children versus that of other children. We then examined the correlation between maternal brain activation and subjective feelings of parenting stress. Brain regions associated with maternal motivation including the orbitofrontal cortex (OFC), ventral pallidum, periaqueductal gray (PAG), dorsal raphe nucleus (DRN), and anterior insular cortex (AIC)—as well as those associated with the recognition of one’s own child’s state (e.g., cerebellum)—exhibited significant activation in response to their own children. While mothers with higher activation in the OFC showed less parenting stress related to one’s sense of competence in the parental role, mothers with higher co-activation of the OFC with both of the AIC and PAG/DRN, and with the cerebellum showed less parenting stress caused by child characteristics. Our findings suggest that well-balanced maternal brain mechanisms integrated by the OFC may provide effective adaptive responses in daily parenting scenarios.

## Introduction

While parents generally find joy in parenting, they find that stress is also a normal part of the parenting experience. Parenting stress refers to a set of processes that lead to aversive psychological and physiological reactions arising from attempts to adapt to the demands of parenthood^1^. Moderate levels of parenting stress, such as those associated with evaluating one’s success in the parental role and perceiving a child’s cues appropriately, exert beneficial effects on maternal motivation^2^. However, high levels of parenting stress often impair warm and responsive parenting, provoke harsh and reactive caregiving, and negatively influence the parent-child relationship, children’s outcomes, and the well-being of the mother^3,4^.

Many mothers are adaptive, deploying successful coping strategies that mitigate the deleterious effects of parenting stress on caregiving^5^. That is, mothers typically find themselves highly motivated to take care of their infants’ needs, and they also find their interactions with their infants during daily parenting to be rewarding, even with stress. Nevertheless, neural mechanisms underlying these adaptive responses in healthy mothers remain unclear. In the present study, we focused on feeding behavior in young children in order to evoke adaptive brain responses associated with daily parenting. Feeding is an essential behavior that supports lifelong healthy eating habits and presents an opportunity for interaction between the mother and child. While mealtime often provides a moment to enjoy food with a child, feeding problems such as selective eating habits, negative affect and negativistic behavior during eating, exceedingly slow eating, and angry outbursts during mealtimes are common concerns during childhood^6^. Indeed, the prevalence of problematic eating and feeding behavior is 25% in infants and young children^7^. Therefore, especially during early childhood, maternal concerns regarding feeding behavior are associated with the feelings of joy, worry, and stress, even if the child is behaving appropriately.

In our previous study, we demonstrated that a limited number of brain regions are specifically associated with maternal love: the right orbitofrontal cortex (OFC), basal ganglia, anterior insular cortex (AIC), and periaqueductal gray (PAG)^8^. These regions belong to a functional network that mediates feelings of subjective pleasure upon seeing a loved one^9^. Additional studies have revealed that this network facilitates maternal behavior toward one’s own child^8,10–15^ in humans. Accordingly, we hypothesized that these regions also play an important role in maternal brain responses during daily parenting scenarios. The OFC and basal ganglia are involved in the dopamine reward system, while the OFC, insula, and PAG are involved in the interoceptive information processing system. Based on our previous suggestion that the right OFC integrates these two systems, serving to motivate maternal behavior^8,11^, we further hypothesized that the right OFC plays a key role in supporting effective adaptive brain responses. In addition, several studies have revealed that chronic and acute stress may impair OFC functions, including emotional regulation and responsiveness to reward-related information, indicating that the OFC may be associated with stress resilience^16,17^. Moreover, the brain regions related to interception such as AIC and PAG play a critical role in stress regulation^18,19^.

In the present study, we utilized functional magnetic resonance imaging (fMRI) to investigate brain activity in healthy mothers of typically developing, 2-to-3-year-old children in response to the feeding behavior of their own children versus that of other children. We then examined the correlation between maternal brain activation and subjective feelings of daily parenting stress. We also investigated whether individual differences in co-activations (scores of the principal component among some brain regions) of the right OFC with the brain regions related to interoception and with those related to parenting stress are correlated with the experience of parenting stress.

## Results

### Subjective ratings of emotions

We compared subjective ratings of the mother’s emotional responses when viewing her own child’s feeding behavior versus that of other children. Rating scores were significantly higher for feelings of love (z = 4.641, *p* = 0.000), motherly feelings (z = −4.635, *p* = 0.000), happiness (z = 4.571, *p* = 0.000), cuteness (z = 4.483, *p* = 0.000), joy (4.551, *p* = 0.000), calmness (z = 4.640, *p* = 0.000), satisfaction (z = 4.641, *p* = 0.000), excitement (z = 4.233, *p* = 0.000), stress (z = 3.224, *p* = 0.001), and worry (z = 3.281, *p* = 0.001) when the mothers viewed clips of their own children. However, no significant differences in feelings of irritation (z = 0.973, *p* = 0.141) or anxiety (z = 1.474, *p* = 0.141) were observed (Table 1).

**Table 1:** Comparisons of the subjective maternal ratings of emotion.

	Own child	Other children	z values	*p* values
	mean ± sd	mean ± sd		
Motherly	5.00 ± 0.00	2.53 ± 0.68	4.64	0.000*
Love	5.00 ± 0.00	2.37 ± 0.46	4.64	0.000*
Cuteness	4.82 ± 0.38	2.96 ± 0.59	4.48	0.000*
Happiness	4.75 ± 0.51	2.05 ± 0.63	4.57	0.000*
Calmness	4.71 ± 0.45	2.46 ± 0.49	4.64	0.000*
Joy	4.64 ± 0.48	2.46 ± 0.72	4.55	0.000*
Satisfaction	4.14 ± 0.69	1.46 ± 0.45	4.64	0.000*
Excitement	3.00 ± 1.10	1.12 ± 0.38	4.23	0.000*
Stress	2.07 ± 1.07	1.20 ± 0.36	3.22	0.001*
Worry	1.93 ± 0.84	1.22 ± 0.37	3.28	0.001*
Anxiety	1.46 ± 0.78	1.21 ± 0.37	1.47	0.141
Irritation	1.25 ± 0.51	1.18 ± 0.36	0.97	0.141

Mean scores of the mothers’ feelings toward their own children and other children in feeding scenario.

1 = completely disagree, 2 = slightly disagree, 3 = slightly agree, 4 = certainly agree, 5 = completely agree.

sd; standard deviation.

*Statistically significant.

Among the 12 descriptors of the mother’s feelings toward their own children’s feeding behavior, scores for love, motherly feelings, happiness, joy, calmness, and satisfaction were significantly higher than those for excitement, stress, worry, irritation, and anxiety.

### Brain activation in response to the feeding behavior of one’s own child

Significant activation was observed in the following regions when mothers viewed their own children’s feeding behavior, relative to that observed when they viewed the feeding behavior of other children: OFC, AIC, PAG/dorsal raphe nucleus (DRN), ventral pallidum (VP), ventrolateral prefrontal cortex (VLPFC), parahippocampal gyrus, intraparietal sulcus, ventral premotor area, superior temporal sulcus (STS), temporo-parietal junction (TPJ)/posterior STS (pSTS), and anterior cerebellum (aCB) in the right hemisphere; TPJ, temporal pole, and middle temporal gyrus (MTG) in the left hemisphere; and fusiform gyrus, posterior cerebellum (pCB), and cerebellar vermis in both hemispheres. No significant activation was observed when mothers viewed other children’s feeding behavior (Table 2).

**Table 2:** Mother’s brain activations in response to child’s eating situation.

Brain regions	MNI coordinates			t values	k
	x	y	z		
**own child > unknown children**					
Orbitofrontal cortex (OFC)	36	18	−16	6.5	7
Periaqueductal gray (PAG)/Dorsal raphe nucleus (DRN)	6	−28	−10	6.8	23
Anterior Insula cortex (AIC)	46	8	2	6.4	12
	40	6	−4	6	1
Ventral pallidum (VP)	20	−10	−2	7	27
Ventrolateral prefrontal cortex (VLPFC)	48	20	16	7.7	312
	46	10	24	7.5	
	48	4	34	6.3	
Ventral premotor area	40	−2	34	6.1	2
	44	0	44	6	2
Intraparietal sulcus	26	−78	30	6.9	24
Middle temporal gyrus (MTG)	−52	−64	4	7	42
Parahippocampal gyrus	24	−30	2	6.7	12
	28	−26	−8	6.4	6
	24	−28	−4	6	1
Temporal pole	−50	10	−20	6.2	3
Superior temporal sulcus (STS)	60	−14	−12	6.3	1
Temporoparietal junction (TPJ)	−58	−32	30	6.7	21
TPJ/Posterior STS (TPJ/pSTS)	54	−58	4	11	2771
Fusiform gyrus	48	−48	−18	13	
	48	−54	−12	12	
	−46	−54	−14	8.1	144
Posterior cerebellum (pCB)	−34	−56	−28	7.4	
	38	−56	−30	6.9	23
Anterior cerebellum (aCB)	24	−42	−38	6.1	1
Cerebellum Vermis	−2	−62	−40	7.3	66
	6	−72	−38	7.1	
**unknown children >own child**					
	n.s.				

The region in the table was significant at the threshold of *p* < 0.05, FWEp; κ, cluster size (number of voxels); MNI, Montreal Neurological Institute; PSI, Parental Stress Index.

### Association between maternal brain responses and daily parenting stress

**Figure 1:**
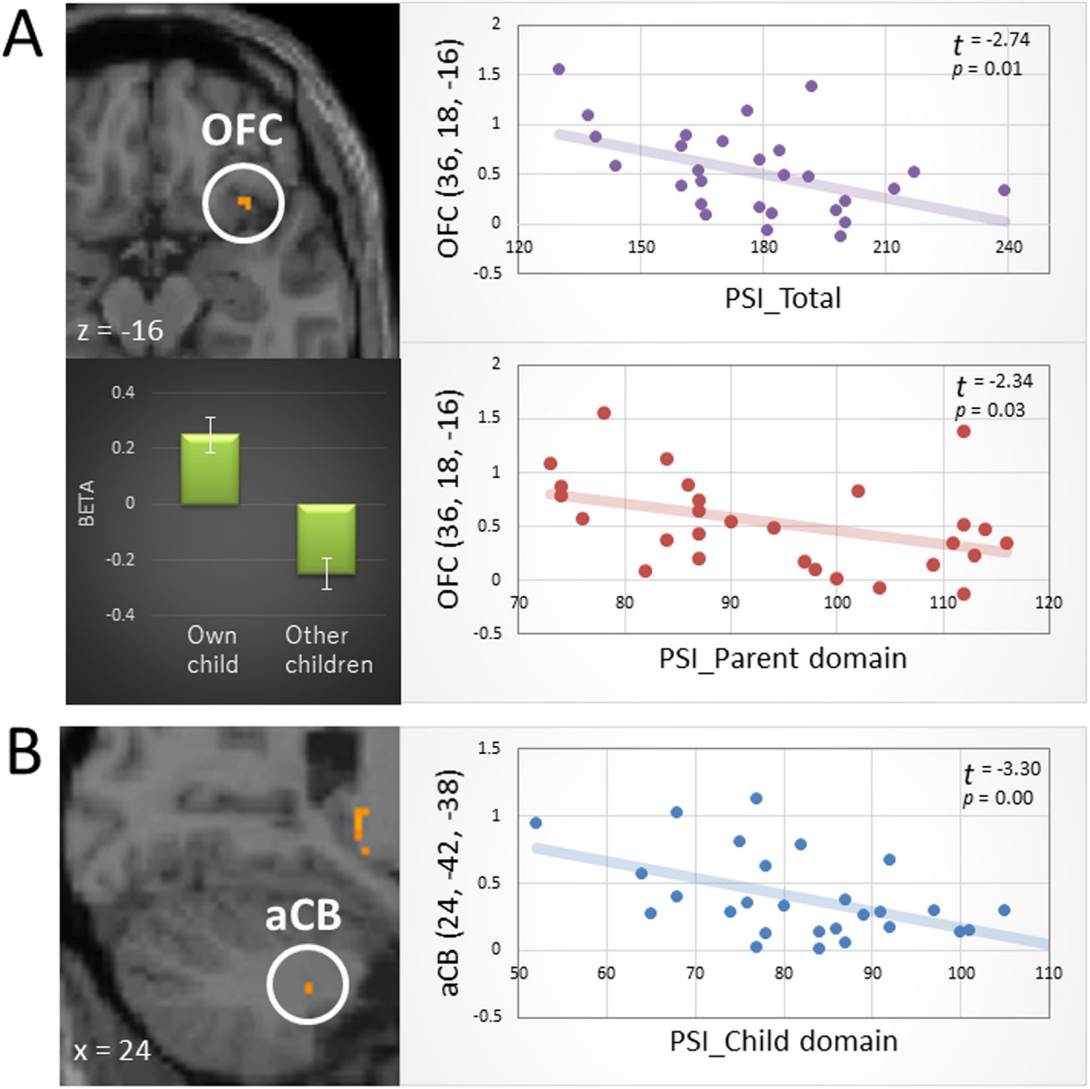
The correlation between the magnitude of activation in the brain regions exhibiting significant activation in response to the mother’s own child compared with other children and the score of Parenting Stress Index (PSI). (**A**) The first eigenvariate of functional region of interest (ROI) in the right orbitofrontal cortex (OFC) showed negative correlations with the total PSI scores (purple dots and line) and with the PSI scores on the parent domain (red dots and line). The bar graph illustrates averages and standard errors of the first eigenvariate of the OFC ROI in the own child vs. baseline and the other children vs. baseline. (**B**) The first eigenvariate of functional ROI in the right anterior cerebellum (aCB) showed negative correlation with the PSI scores on the child domain.

Among the regions exhibiting significant activation when mothers viewed the feeding behavior of their own children, the magnitude of activation in the right OFC was negatively correlated with the total PSI scores (adjusted R^2^ = 0.194; t = −2.741, *p* = 0.011; S-W statistic = 0.947, *p* = 0.162; D-W statistic = 1.796) and with scores on the parent domain (adjusted R^2^ = 0.142; t = −2.341, *p* = 0.027; S-W statistic = 0.944, *p* = 0.144; D-W statistic = 2.090) (Fig. 1A). In contrast, activation in the aCB was negatively correlated with scores on the child domain (adjusted R^2^ = 0.272; t = −3.330, *p* = 0.003; S-W statistic = 0.980, *p* = 0.847; D-W statistic = 1.907) (Fig. 1B).

**Figure 2:**
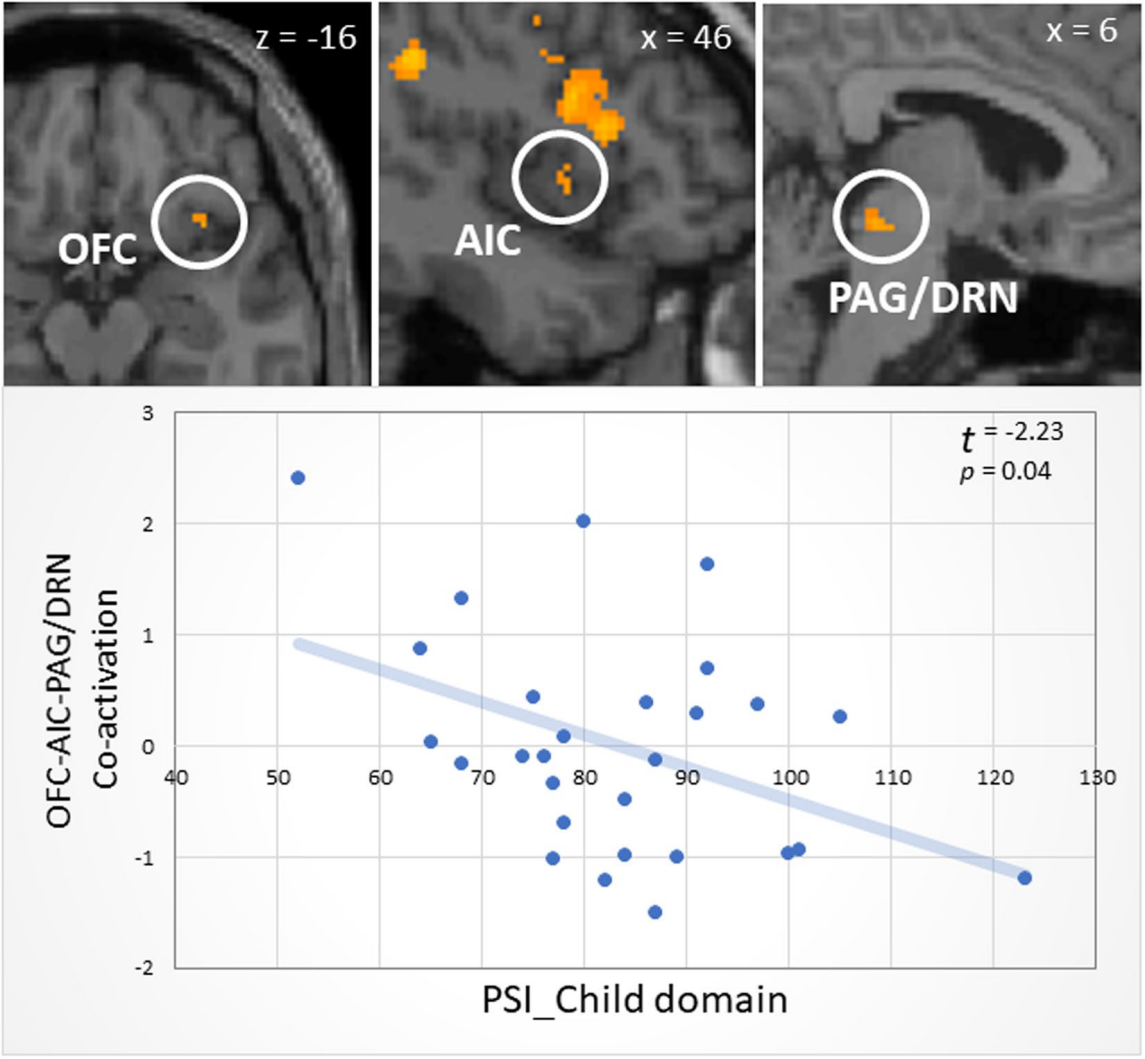
The correlation between the score of Parenting Stress Index (PSI) and co-activation index among the brain regions associated interoception. Co-activation index among the right orbitofrontal cortex (OFC), anterior insula cortex (AIC), and periaqueductal gray/dorsal raphe nucleus (PAG/DRN) was negatively correlated with scores on the child domain.

In addition, we extracted the first principal component among the OFC, insula and PAG (68.196% of the total variance) of which principal component loadings were 0.927, 0.782 and 0.758, and score coefficients were 0.453, 0.370 and 0.382, respectively. We confirmed the sampling adequacy (KMO measure = 0.536). Moreover, the principal component scores were negatively correlated with scores on the child domain (adjusted R^2^ = 0.147; t = −2.376, *p* = 0.025; S-W statistic = 0.952, *p* = 0.220; D-W statistic = 2.345) (Fig. 2). In addition, we extracted the first principal component between the OFC and aCB (75.608% of the total variance), and their principal component loadings and the score coefficients were 0.870 and 0.575, respectively. We confirmed the sampling adequacy (KMO measure = 0.520). Moreover, the principal component scores were negatively correlated with scores on the child domain (adjusted R^2^ = 0.279; t = −3.387, *p* = 0.002; S-W statistic = 0.978, *p* = 0.807; D-W statistic = 2.517).

Mean scores of the total PSI was 177.78 ± 24.66, parent domain was 94.39 ± 13.88 and child domain was 83.29 ± 14.16.

## Discussion

In the present study, we investigated brain activation in healthy mothers in response to the feeding behavior of their own children, versus that of other children. Our results indicated that the intensity of positive feelings was significantly higher when mothers viewed video clips of their own children eating than when they viewed clips of other children eating. In addition, the intensity of negative feelings (worry and stress) was significantly higher when viewing mother’s own child than other children. These results suggest that feelings of worry and stress underlie parenting stress, even when the mother reports clear positive feelings regarding her own child. Accordingly, automatic regulation of negative feelings is important for facilitating and maintaining maternal motivation in daily parenting scenarios. In this study, specific positive feelings were elicited when the mother viewed her own child’s eating behavior, suggesting that the mothers demonstrated effective adaptive brain responses required for daily parenting, including maternal warmth and potential stress, during the fMRI examination.

In accordance with our previous findings^8^, mothers in the present study exhibited significant activation in brain regions associated with maternal love in response to viewing their own children’s feeding behavior: the right OFC, VP, PAG/DRN, and AIC. These regions may play an important role in creating maternal motivation for caretaking in daily life^8,20^. The OFC plays an important role in the reward system and is associated with higher-order dimensions of maternal love^8,11–13,15,20^. In addition, the OFC receives ascending dopamine projections from the VTA and is critical in representing the value of a stimulus reward^21,22^. Indeed, previous studies have demonstrated the importance of dopamine signaling in maternal brains using dopamine transporter-knockout mice, which exhibited impaired maternal behavior^23,24^. Interestingly, right OFC activation was negatively correlated with total PSI scores and scores on the parent domain, but not with those on the child domain. Higher scores on the parent domain reflect potential dysfunction regarding the mother’s sense of parenting competence and her own functioning in the parental role, suggesting that right OFC activation is associated with the mother’s sense of parenting competence. Such activation may in turn be necessary for maintaining maternal motivation and decreasing overall parenting stress. An fMRI study using emotional infant stimuli reported that increased cortisol reactivity was associated with poor maternal functioning, but not necessarily a distressed infant^25^. This finding suggests that the maternal motivation involved in OFC activation is affected by stress in the parent domain, but not by stress in the child domain. Thus, the OFC may mediate reward processing for not only the mother’s own infant but also the mother’s own functioning within the family.

The co-activation among the right OFC, AIC and PAG/DRN were significantly negatively correlated with parenting stressors in the child domain, although independent activation in each of these regions was not significantly correlated with parenting stress. These results indicate that the right OFC may modulate parenting stress in the child domain by cooperating with the brain regions involved in interoception.

Previous human neuroimaging studies have demonstrated that the insular cortex plays an important role in the maternal brain^8,13,20,26–29^. The posterior insular cortex has been commonly associated with primary interoceptive representation of the physiological condition of the body^30^, whereas the AIC participates in a wide range of functions related to interoceptive, emotional, and empathetic awareness^31,32^. Recent imaging studies have reported that activation of the AIC—which is interconnected with the OFC^33,34^—occurs during subjective emotional experiences (e.g., maternal love)^8,20,28,35^. Laurent and colleagues^25^ also suggest that the activation of the AIC and OFC in response to a potentially stressful parenting context is associated with more effective stress regulation in mothers with greater empathic attunement. Furthermore, a meta-representation of primary interoceptive activity is engendered in the right anterior insula, which seems to provide the basis for the subjective image of the material self as a feeling entity (i.e., emotional awareness)^36^. A previous study has reported that the right insular cortex plays a greater role than the left in supporting reward evaluation and motivational approaches to positive stimuli^37^. An additional study has noted that the perceived warmth of a mother’s infant was correlated with activation in her right AIC when viewing her infant^33^. These findings indicate that activation of the right AIC may mediate the mother’s understanding of what her child is feeling internally and allow her to realize her own motherhood.

The PAG, which mainly projects from the insula and prefrontal cortex, plays a pivotal role in the integration of the emotional aspects of homeostatic regulation via the autonomic nervous system^38^. The PAG also exhibits direct connections with the OFC^39,40^ which plays an important role in orchestrating maternal behaviors across mammalian species^10,41,42^. Additionally, several fMRI studies have suggested that the PAG is specifically involved in human maternal behaviors, such as representing the end goal of a well-regulated maternal response to infant cues^8,20,25,43^. Furthermore, the PAG contains a high density of oxytocin receptors^44^, which are important for the onset and maintenance of perinatal maternal behavior^13,45–50^, and also mediate feelings of empathy and stress reduction^51,52^. Other research groups have observed that oxytocin in the PAG plays a key role in maternal behavior by modulating anxiety-related behaviors in mother rats^53,54^. These findings indicate that the PAG may also play an important role in facilitating and maintaining empathetic maternal behavior in humans.

The DRN—a serotonin-rich site—exhibits well-established interconnections with the OFC. Serotonin is a neurotransmitter that plays an essential role in the regulation of emotion. In addition, stress is known to alter serotonin receptors in the DRN^55^. Kranz *et al*. have argued that serotonin represents a fundamental mediator of the emotional, motivational, and cognitive aspects of reward representation^56^. That is, the DRN serves as an important reward-processing station in parallel to the midbrain dopamine center^57^. Kikuchi and colleagues^58^ observed that DRN activation was negatively correlated with attachment-related anxiety in healthy adults. These findings suggest that DRN activation may be associated with maternal feelings of attachment towards one’s own child.

In accordance with previous findings, several brain regions exhibited common activation when mothers viewed their own children, but did not exhibit activation when they viewed other children: the VLPFC, ventral premotor area, MTG, parahippocampal gyrus, temporal pole, TPJ/pSTS, fusiform gyrus, cerebellum, and cerebellar vermis. These regions are involved in the representation of the child’s intentions and mental states, and are critical for mother-child interactions/human parental care^10–14^. In particular, activation in the anterior cerebellum was negatively correlated with parenting stress in the child domain. Higher scores on the child domain mainly reflect difficulty in the parental role due to the characteristics of the child. Several fMRI studies have reported that cerebellar activation is associated with empathy and mentalization^59–61^. A meta-analysis by Van Overwalle *et al*.^60^ suggested that the aCB is involved in processing the enduring characteristics of other individuals (e.g., traits or preferences), while the pCB is associated with the momentary intentions, beliefs, and mental states of others. These findings indicate that activation in the aCB of the mother’s brain may facilitate inferences regarding her child’s characteristics, which may act to decrease stress in the child domain. Furthermore, we observed that parenting stress in the child domain was significantly negatively correlated with co-activation in the right OFC-aCB. These findings suggest that motivation to understand the characteristics of one’s own child to reduce parenting stress is mediated by the right OFC.

Taken together, our results suggest that the OFC may play a central role in providing intrinsic maternal motivation and may be critical for adaptation in daily parenting situations. In contrast, dysfunction of the right OFC may impair maternal motivation and the realization of one’s own motherhood, which may produce subjective feelings of poor maternal functioning and make it difficult for a mother to understand her child’s needs. The findings of the present study suggest that well-balanced maternal brain mechanisms integrated by the OFC may engender resilience to parenting stress and promote subjective maternal experiences (e.g., “I love my child dearly,” “I am alive through my child,” “I want my child to grow up happy and healthy”). Such responses likely aid in maintaining maternal motivation and regulating maternal stress adaptation during the “terrible twos.”

## Methods and Materials

### Participants

Twenty-eight healthy, right-handed, first-time mothers (age: 35.14 ± 4.86 years) of young children (age: 31.89 ± 6 .12 months; 13 boys and 15 girls; all healthy firstborns) participated in the present study. Mothers had no history of major physical or mental illness, none were taking any medications at the time of the study, and none were pregnant. All mothers underwent psychological assessment using the General Health Questionnaire 28^62^ (GHQ28; total scale: 4.39 ± 3.18, somatic symptoms: 2.00 ± 1.58, anxiety/insomnia symptoms: 2.00 ± 1.22, social dysfunction: 0.32 ± 1.14, severe depression: 0.07 ± 0.26). All participants provided written and oral informed consent to participate in this study for themselves and their children. The present study was approved by the Research Ethics Committee of Tokyo Metropolitan University and the Research Ethics Committee of the National Center of Neurology and Psychiatry, Japan. All methods were performed in accordance with the relevant guidelines and regulations.

### Procedure

The experimental stimuli were created by recording the feeding behavior of each mother’s child on video at Tokyo Metropolitan University approximately 1–2 months before the fMRI session. Light snacks were used to avoid the influence of selective eating behavior during the session. All children sat in the same infant chair in front of a gray wall. Each child ate some snacks and drank a beverage, which were chosen by his/her mother and placed on a table. The video camera was positioned approximately 5.25 feet across the table from the child and next to the mother. Each video recording lasted about 15 min, and four different clips, each with duration of 30 s, were edited from each recording using Adobe Premiere Elements 10 (Adobe Systems Incorporated, San Jose, CA). In each clip, the child’s face and upper chest were visible in a circle on a black background.

### Experimental stimuli

During the fMRI session, each mother viewed the video clips of her own child’s feeding behavior, as well as those of four other children unknown to her. Each trial consisted of eight task blocks, each lasting 30 s: Four clips of the mother’s own child and four clips of four other unknown children were presented in random order. The sex and age of the children were counterbalanced. Before each task block, a white cross was displayed on a black screen for 12 s (rest block). The video stimuli were presented with no sound due to the high variance in auditory information. The mothers were instructed to view the video clips projected from behind the screen through mirrors, with the circle occupying a visual angle of approximately 12.2° × 12.2°.

### Acquisition of fMRI data

All images were obtained using a 3 T MRI scanner (Achieva Series Quasar Dual 3.0 T; Philips Medical Systems, Best, Netherlands). Whole-brain functional images were acquired using a T2*-weighted echo-planar imaging (EPI) sequence with 25 slices (thickness: 5.0 mm) of 128 × 128 pixels. Additional parameters were as follows: echo time (TE) = 35 ms, repetition time (TR) = 3,000 ms, field-of-view (FOV) = 230 mm, flip angle (FA) = 90°, dynamic scans = 116 volumes. T1-weighted anatomical images were acquired using the following parameters: 150 slices (thickness: 1.0 mm), TE = 1.99 ms, TR = 23 ms, FOV = 258 mm, FA = 8°, matrix size = 288.

### Post-imaging emotional assessments

After the fMRI scan, the mother was asked to view sample video clips and rate her feelings. The sample video clips were selected from among the stimuli that had been presented to her while in the fMRI scanner and consisted of one clip of the mother’s own child and four of other children. The mother was asked to rate each of 12 descriptors (happiness, joy, motherly feelings, feelings of love, calmness, satisfaction, cuteness, excitement, irritation, anxiety, worry, and stress) on a five-point scale (1 = completely disagree, 2 = slightly disagree, 3 = slightly agree, 4 = certainly agree, 5 = completely agree). Differences in ratings between the mother’s feelings toward her own child and other children (i.e., the average rating across the other four children) were compared using the Wilcoxon signed rank test based on Bonferroni correction with a significance level of p < 0.05. Each mother’s ratings of the 12 descriptors for her own child were compared using the Friedman’s test and post-hoc tests with a significance level of p < 0.05 via Bonferroni correction.

### Measures of parenting stress

To investigate the relationship between brain activation and daily parenting stress, we used the Japanese version of the Parental Stress Index (PSI)^63^, a 78-item self-report questionnaire based on the original version developed by Abidin^63,64^, the validity and reliability of which have been confirmed. These 78 items were divided into 15 subscales across two major domains: child and parent. Stressors in the child domain include the child’s temperamental and behavioral characteristics (e.g., high irritability and difficulty with compliance), while those in the parent domain include aspects of parental functioning and personality components, such as feelings of guilt, depression, and a sense of low competence in the parental role. Higher scores on the parent domain reflect potential dysfunction with respect to one’s sense of parenting competence and the respondent’s own functioning within the family. Higher scores on the child domain reflect difficulty in the parental role due to child characteristics. Participants rated the items on a five-point scale, ranging from 1 (completely disagree) to 5 (completely agree). Higher total scores indicate higher levels of parenting stress. Score of 90, 105, and 121 on the parent domain reflect percentile scores of 20, 50, and 80, respectively. Score of 74, 86, and 98 on the child domain reflect percentile scores of 20, 50, and 80, respectively^63^. The PSI was measured after rating mother’s feelings toward sample video clips.

### Analysis of fMRI data

The fMRI data of each participant were analyzed separately using SPM8 (http://www.fil.ion.ucl.ac.uk/spm/software/spm8/). The images were spatially realigned, co-registered (T1 to EPI), normalized to the Montreal Neurological Institute (MNI) template, and smoothed using a Gaussian filter of 8-mm full-width at half maximum. The data were temporally convolved with the hemodynamic response function (HRF) and high-pass filtered with a cutoff period of 128 s. Each feeding scenario (own infant, other infants) was modeled using a separate regressor for each participant, and the contrasts between brain activity for the mother’s own child and other children were examined. Random effects analysis was then performed at a family-wise error (FWEp, peak-level)-corrected level of *p* = 0.05.

### Associations between maternal brain responses and daily parenting stress

Each cluster that exhibited significant activation in response to the mother’s own child when compared with other children was regarded as a functional region of interest (ROI). The first eigenvariate of each ROI in each mother was used for regression analyses. Multiple stepwise regression analyses were conducted to examine the association between each of PSI scores (total, parent, and child) as the dependent variable and the first eigenvariate in all the ROIs as the independent variables.

Moreover, we calculated an individual’s co-activation among the OFC, insula and PAG, and between the OFC and the other ROI which showed a significant correlation with each PSI, by computing the principal component scores via principal component analyses, based on the second-level analysis. In addition, we checked the sampling adequacy by using the Kaiser-Meyer-Olkin (KMO) test. We then conducted stepwise multiple regression analyses using each principal component score as the dependent variable and PSI scores on parent and child domains as independent variables.

The level of significance for all regression analyses was corrected using the Holm method (p < 0.05). We then assessed the residuals for all regression analyses by using the S-W test of normality (p < 0.05), and calculated the D-W statistic for the null hypothesis of no autocorrelation. Statistical analyses were performed using SPSS version 20.0 software (SPSS, INC., Chicago, IL).

## References

[1] Deater-Deckard, K. *Parenting Stress*. New Haven, CT (Yale University Press, 2004).

[2] Henderson RK, Snyder HR, Gupta T, Banich MT (2012). When does stress help or harm? The effects of stress controllability and subjective stress response on stroop performance. Front Psychol..

[3] Lupien SJ, McEwen BS, Gunnar MR, Heim C (2009). Effects of stress throughout the lifespan on the brain, behaviour and cognition. Nat Rev Neurosci..

[4] Chan JC, Nugent BM, Bale TL (2018). Parental Advisory: Maternal and Paternal Stress Can Impact Offspring Neurodevelopment. Biol Psychiatry..

[5] Deater-Deckard, K., Chen, N., & Mallah, S. E. *Parenting Stress* (Oxford Biolographies, 2015).

[6] Lewinsohn PM (2005). Problematic eating and feeding behaviors of 36-month-old children. Inf Eat Disord..

[7] Manikam R, Perman JA (2000). Pediatric feeding disorders. J Clin Gastroenterol..

[8] Noriuchi M, Kikuchi Y, Senoo A (2008). The functional neuroanatomy of maternal love: mother’s response to infant’s attachment behaviors. Biol Psychiatry..

[9] Berridge KC, Kringelbach ML (2015). Pleasure systems in the brain. Neuron..

[10] Barrett J, Fleming AS (2011). Annual Research Review: All mothers are not created equal: neural and psychobiological perspectives on mothering and the importance of individual differences. J Child Psychol Psychiatry..

[11] Kikuchi, Y. & Noriuchi, N. Neural basis of maternal love as a vital human emotion. *In: Fukuda S*, *editor*. *Emotional Engineering volume 4* (Springer, pp 189–198, 2016).

[12] Swain JE (2014). Approaching the biology of human parental attachment: brain imaging, oxytocin and coordinated assessments of mothers and fathers. Brain Res..

[13] Rilling JK (2013). The neural and hormonal bases of human parental care. Neuropsychologia..

[14] Kim P, Strathearn L, Swain JE (2016). The maternal brain and its plasticity in humans. Horm Behav..

[15] Zeki S (2007). The neurobiology of love. FEBS Lett..

[16] Varga Z (2017). Chronic stress affects the number of GABAergic neurons in the orbitofrontal cortex of rats. Behavioural Brain Research..

[17] Porcelli AJ, Lewis AH, Delgado MR (2012). Acute stress influences neural circuits of reward processing. Front Neurosci..

[18] Schulz A, Vögele C (2015). Interocaption and stress. Front Psychol..

[19] Barrett LF, Simmons WK (2015). Interoceptive predictions in the brain. Nat Rev Neurosci..

[20] Bartels A, Zeki S (2004). The neural correlates of maternal and romantic love. Neuroimage..

[21] Schoenbaum G, Chiba AA, Gallagher M (1998). Orbitofrontal cortex and basolateral amygdala encode expected outcomes during learning. Nat Neurosci..

[22] O’Doherty J (2001). Abstract reward and punishment representations in the human orbitofrontal cortex. Nat Neurosci..

[23] Spielewoy C (2000). Behavioural disturbances associated with hyperdopaminergia in dopamine-transporter knockout mice. Behav Pharmacol..

[24] Kavelaars A, Cobelens PM, Teunis MA, Heijnen CJ (2005). Changes in innate and acquired immune responses in mice with targeted deletion of the dopamine transporter gene. J Neuroimmunol..

[25] Laurent HK, Stevens A, Ablow JC (2011). Neural correlates of hypothalamic-pituitary-adrenal regulation of mothers with their infants. Biol Psychiatry..

[26] Kim P (2011). Breastfeeding, brain activation to own infant cry, and maternal sensitivity. J Child Psychol Psychiatry..

[27] Lenzi D (2009). Neural basis of maternal communication and emotional expression processing during infant preverbal stage. Cereb Cortex..

[28] Atzil S (2012). Synchrony and specificity in the maternal and the paternal brain: relations to oxytocin and vasopressin. J Am Acad Child Adolesc Psychiatry..

[29] Swain JE (2012). Parenting and beyond: common neurocircuits underlying parental and altruistic caregiving. Parent Sci Pract..

[30] Craig AD (2002). How do you feel? Interoception: the sense of the physiological condition of the body. Nat Rev Neurosci..

[31] Craig AD (2009). How do you feel–now? The anterior insula and human awareness. Nat Rev Neurosci..

[32] Gu X, Hof PR, Friston KJ, Fan J (2013). Anterior insular cortex and emotional awareness. J Comp Neurol..

[33] Craig AD (2009). Emotional moments across time: a possible neural basis for time perception in the anterior insula. Philos Trans R Soc Lond B Biol Sci..

[34] Bernhardt BC, Singer T (2012). The neural basis of empathy. Annu Rev Neurosci..

[35] Wan MW (2014). The neural basis of maternal bonding. PLoS One..

[36] Craig AD (2003). Interoception: the sense of the physiological condition of the body. Curr Opin Neurobiol..

[37] Vijayaraghavan L (2013). A selective role for right insula–basal ganglia circuits in appetitive stimulus processing. Soc Cogn Affect Neurosci..

[38] Linnman C (2011). Neuroimaging of the periaqueductal gray: state of the field. Neuroimage..

[39] Cavada C (2000). The anatomical connections of the macaque monkey orbitofrontal cortex. A review. Cereb Cortex..

[40] Jenkins JS (1984). Vasopressin, oxytocin and neurophysins in the human brain and spinal cord. Brain Res..

[41] Sukikara MH (2010). The periaqueductal gray and its potential role in maternal behavior inhibition in response to predatory threats. Behav Brain Res..

[42] Klein MO (2014). Periaqueductal gray mu and kappa opioid receptors determine behavioral selection from maternal to predatory behavior in lactating rats. Behav Brain Res..

[43] Laurent HK, Ablow JC (2012). A cry in the dark: depressed mothers show reduced neural activation to their own infant’s cry. Soc Cogn Affect Neurosci..

[44] Jenkins JS (1984). Vasopressin, oxytocin and neurophysins in the human brain and spinal cord. Brain Res..

[45] Kendrick, K. M. Oxytocin, motherhood and bonding. *Exp Physiol*. **85**, Spec No:111S-124S (2000). 10.1111/j.1469-445x.2000.tb00014.x 10795913

[46] Baskerville TA, Douglas AJ (2010). Dopamine and oxytocin interactions underlying behaviors: potential contributions to behavioral disorders. CNS Neurosci Ther..

[47] Finkenwirth C, Martins E, Deschner T, Burkart JM (2016). Oxytocin is associated with infant-care behavior and motivation in cooperatively breeding marmoset monkeys. Horm Behav..

[48] Gordon I, Zagoory-Sharon O, Leckman JF, Feldman R (2010). Oxytocin and the development of parenting in humans. Biol Psychiatry..

[49] Feldman R (2012). Sensitive parenting is associated with plasma oxytocin and polymorphisms in the OXTR and CD38 genes. Biol Psychiatry..

[50] Kim S, Fonagy P, Koos O, Dorsett K, Strathearn L (2014). Maternal oxytocin response predicts mother-to-infant gaze. Brain Res..

[51] Carrasco GA, V de Kar LD (2003). Neuroendocrine pharmacology of stress. Eur J Pharmacol..

[52] De Dreu CK (2012). Oxytocin modulates cooperation within and competition between groups: an integrative review and research agenda. Horm Behav..

[53] Figueira RJ, Peabody MF, Lonstein JS (2008). Oxytocin receptor activity in the ventrocaudal periaqueductal gray modulates anxiety-related behavior in postpartum rats. Behav Neurosci..

[54] MacDonald K, Feifel D (2014). Oxytocin’s role in anxiety: A critical appraisal. Brain Res..

[55] Fuchs E, Flügge G (2003). Chronic social stress: effects on limbic brain structures. Physiol Behav..

[56] Kranz GS, Kasper S, Lanzenberger R (2010). Reward and the serotonergic system. Neuroscience..

[57] Li Y (2016). Serotonin neurons in the dorsal raphe nucleus encode reward signals. Nat Commun..

[58] Kikuchi Y (2017). Brainstem activity predicts attachment-related anxiety. Neuropsychiatry..

[59] Hillis AE (2014). Inability to empathize: brain lesions that disrupt sharing and understanding another’s emotions. Brain..

[60] Van Overwalle F, Baetens K, Mariën P, Vandekerckhove M (2014). Social cognition and the cerebellum: a meta-analysis of over 350 fMRI studies. Neuroimage..

[61] Seehausen M (2016). Effects of empathic social responses on the emotions of the recipient. Brain Cogn..

[62] Goldberg, D. P. *Manual of the General Health Questionnaire* (Sussex: DJS Spools, 1978).

[63] Narama M (1999). Validity and Reliability of the Japanese Version of the Parenting Stress Index. The journal of child health..

[64] Abidin, R. R. *Parenting Stress Index Professional Manual (3rd ed.)*. Odessa, FL: Professional Assessment Resources (1995).

